# The spatial and temporal scales of local dengue virus transmission in natural settings: a retrospective analysis

**DOI:** 10.1186/s13071-018-2662-6

**Published:** 2018-02-02

**Authors:** Luigi Sedda, Ana Paula Pessoa Vilela, Eric Roberto Guimarães Rocha Aguiar, Caio Henrique Pessoa Gaspar, André Nicolau Aquime Gonçalves, Roenick Proveti Olmo, Ana Teresa Saraiva Silva, Lízia de Cássia da Silveira, Álvaro Eduardo Eiras, Betânia Paiva Drumond, Erna Geessien Kroon, João Trindade Marques

**Affiliations:** 1 Centre for Health Information Computation and Statistics (CHICAS), Furness Building, Lancaster University, Lancaster, LA1 4YG UK; 2 Department of Biochemistry and Immunology, Instituto de Ciências Biológicas, Universidade Federal de Minas Gerais, Belo Horizonte, Minas Gerais 30270-901 Brazil; 3 Department of Microbiology, Instituto de Ciências Biológicas, Universidade Federal de Minas Gerais, Belo Horizonte, Minas Gerais 30270-901 Brazil; 4 Present Address: Instituto de Ciências da Saúde, Universidade Federal da Bahia, Salvador, Bahia 40110-100 Brazil; 5 Department of Parasitology, Instituto de Ciências Biológicas, Universidade Federal de Minas Gerais, Belo Horizonte, Minas Gerais 30270-901 Brazil

**Keywords:** *Aedes aegypti*, *Aedes albopictus*, Dengue virus serotypes 1 and 3, Bivariate point-process, Kriging, Geostatistical additive models, Urban dengue

## Abstract

**Background:**

Dengue is a vector-borne disease caused by the dengue virus (DENV). Despite the crucial role of *Aedes* mosquitoes in DENV transmission, pure vector indices poorly correlate with human infections. Therefore there is great need for a better understanding of the spatial and temporal scales of DENV transmission between mosquitoes and humans. Here, we have systematically monitored the circulation of DENV in individual *Aedes* spp. mosquitoes and human patients from Caratinga, a dengue endemic city in the state of Minas Gerais, in Southeast Brazil. From these data, we have developed a novel stochastic point process pattern algorithm to identify the spatial and temporal association between DENV infected mosquitoes and human patients.

**Methods:**

The algorithm comprises of: (i) parameterization of the variogram for the incidence of each DENV serotype in mosquitoes; (ii) identification of the spatial and temporal ranges and variances of DENV incidence in mosquitoes in the proximity of humans infected with dengue; and (iii) analysis of the association between a set of environmental variables and DENV incidence in mosquitoes in the proximity of humans infected with dengue using a spatio-temporal additive, geostatistical linear model.

**Results:**

DENV serotypes 1 and 3 were the most common virus serotypes detected in both mosquitoes and humans. Using the data on each virus serotype separately, our spatio-temporal analyses indicated that infected humans were located in areas with the highest DENV incidence in mosquitoes, when incidence is calculated within 2.5–3 km and 50 days (credible interval 30–70 days) before onset of symptoms in humans. These measurements are in agreement with expected distances covered by mosquitoes and humans and the time for virus incubation. Finally, DENV incidence in mosquitoes found in the vicinity of infected humans correlated well with the low wind speed, higher air temperature and northerly winds that were more likely to favor vector survival and dispersal in Caratinga.

**Conclusions:**

We have proposed a new way of modeling bivariate point pattern on the transmission of arthropod-borne pathogens between vector and host when the location of infection in the latter is known. This strategy avoids some of the strong and unrealistic assumptions made by other point-process models. Regarding virus transmission in Caratinga, our model showed a strong and significant association between high DENV incidence in mosquitoes and the onset of symptoms in humans at specific spatial and temporal windows. Together, our results indicate that vector surveillance must be a priority for dengue control. Nevertheless, localized vector control at distances lower than 2.5 km around premises with infected vectors in densely populated areas are not likely to be effective.

**Electronic supplementary material:**

The online version of this article (10.1186/s13071-018-2662-6) contains supplementary material, which is available to authorized users.

## Background

Dengue is a mosquito-borne viral infection that affects 500 million people every year [1]. Considering the large number of infections, dengue causes significant mortality and morbidity worldwide [2, 3]. Three quarters of human infections are estimated to be asymptomatic but a small percentage of patients can develop a severe and deadly form of dengue [1]. According to the World Health Organization, Brazil currently occupies the first place in the ranking of reported dengue cases in the world with incidence rates increasing since 2004 [4].

Dengue virus (DENV) is a positive single-stranded RNA virus belonging to the genus *Flavivirus* of the family *Flaviviridae* [5]. There are at least four distinct types of DENV that are genetically related by sharing at least 60% sequence similarity (here simply denoted as DENV1, DENV2, DENV3 and DENV4) [6], and co-circulating in several parts of the world including Brazil [7–9].

The DENV transmission cycle involves mosquitoes and humans, although virus circulation in other vertebrate hosts has also been reported [10, 11]. *Aedes aegypti* mosquitoes are mostly responsible for the urban transmission cycle [3] while *Ae. albopictus* have been suggested to have a role communicating sylvatic and urban cycles of DENV [12, 13]. Despite the essential role of mosquitoes in DENV transmission, there is little correlation between vector indices and human outbreaks [14–17] and hence the importance of this study. DENV transmission is highly dependent on environmental variables that influence vector population dynamics and virus-vector interactions. Macro-scale studies indicated that temperature, wind speed and precipitation are related to changes in DENV incidence (reviewed in [18]). Vector survival depends on water availability and therefore on precipitation, since larval stages of the mosquito are aquatic [19]. Wind influences flight-related activities of vector mosquitoes such as host-seeking. In fact, it has been shown that wind speed above 0.9 m/s seems to discourage nectar-feeding and biting activity [18]. Biting activity, survival rate and extrinsic incubation period (EIP) of the virus in the insect vector are all temperature-dependent. Higher temperatures reduce the duration of the EIP, increasing the likelihood of DENV transmission [20]. However, DENV transmission can only happen if there is contact between humans and mosquitoes, which depends on host-seeking activities of the vector as well as human mobility [21]. In particular, *Ae. aegypti* is considered a localized vector, with low dispersal range, i.e. around 60 m or less, and rarely beyond 500 m [22–25]. On this basis, *Ae. aegypti* females are expected to visit no more than two or three houses in their lifetime thus creating local clusters of DENV infections [26]. Finally, Stoddard et al. [27] found that house-to-house human mobility (social connections) played a key role in defining individual infection risk. Therefore, the contact rate between humans and infected mosquitoes, and the ecological, socio-economic and cultural factors must be considered when inferring the spatial and temporal scales of DENV transmission [28]. However, most of the studies on DENV transmission have focused only on one dimension, either the spatial scale of mosquito distribution or the temporal range of the transmission. Thus, the spatio-temporal dynamics of DENV transmission between mosquitoes and humans in natural and urban settings remains largely unknown. Nevertheless more effective public health policies require that we understand the scales of DENV circulation, which are essential to help predict and prevent outbreaks by vector control strategies [29]. These are at the moment the only effective intervention against outbreaks due to the absence of efficient treatments and vaccines for DENV.

The aim of this study was to estimate (i) the spatial and temporal scales of local dengue transmission in an urban area, and (ii) the effect of the environmental factors. For the first objective, we first analyzed DENV presence in individual mosquitoes and human patients. Then, in order to estimate the values and significance of the spatial and temporal scales of DENV infections in mosquitoes and humans, we developed a bivariate spatio-temporal model. This combines local ordinary kriging (a common interpolator, often used to predict the distribution, dispersal, and abundance of mosquito vectors [22]) of DENV incidence in mosquitoes and permutation of the locations of human DENV cases. For the second aim, we applied a geostatistical additive linear model [30] to identify the important environmental factors that correlated with DENV incidence in mosquitoes found in the proximity of human patients. Importantly, our model does not require the restrictive assumptions made by other spatio-temporal point processes such as marked-point process or inhomogeneous Poisson point process [31]. Hence, we have proposed and implemented a novel methodological framework that will help our understanding on the ecology and scales of local DENV transmission in natural settings.

## Results

### Mosquito collection and analysis

**Fig. 1:**
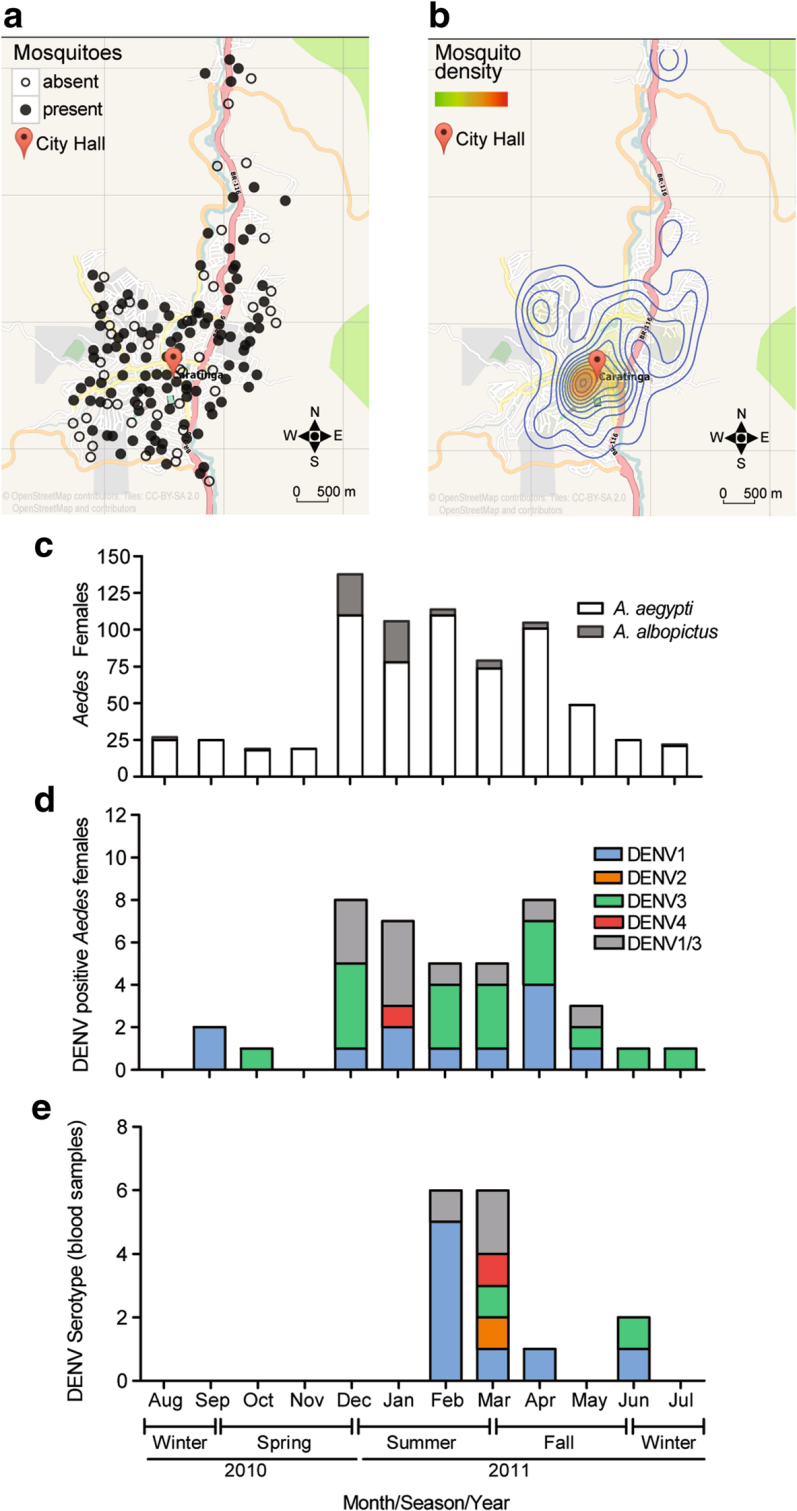
Spatial and temporal distribution of *Aedes* mosquitoes in the city of Caratinga. **a** Localization of mosquito traps in the urban area of Caratinga. Main roads are in yellow, orange and red whereas local streets are in white. Areas shaded in grey are residential and green marks are major forests (source: OpenStreetMap). Filled circles represent traps with at least one mosquito capture. **b** Same map as in **a** showing the density of *Ae. aegypti* and *Ae. albopictus* mosquitoes based on the number of captures in Caratinga. Mosquito density follows the color scale shown in the figure. **c** Total number of female *Aedes* mosquitoes (*Ae. aegypti* and *Ae. albopictus*) captured during the period of the study. Mosquito numbers show an increase in the summer and remain high until the fall. **d** Number of DENV infected *Aedes* females captured during this study where virus types are indicated by color. **e** Number of human dengue cases that were positive for viral RNA in the blood with the DENV types indicated by color

Between August 2010 and July 2011, we performed a comprehensive spatiotemporal survey of mosquitoes in the city of Caratinga utilizing 158 mosquito traps evenly distributed throughout the urban area (Fig. 1a). *Aedes* spp. females represented 95.9% of 763 mosquitoes captured, which confirms the preference described for the traps we utilized [32]. We observed higher numbers of *Ae. aegypti* (655) females compared to *Ae. albopictus* (73) as previously observed for urban areas [33–36]. We also captured a small number of male *Aedes* mosquitoes (30 *Ae. aegypti* and one *Ae. albopictus*) and four *Culex* mosquitoes. These results are in accordance with the types of traps we utilized that were designed for highly specific and detailed surveillance of *Aedes* mosquitoes and not population control [29, 37]. Spatially, *Aedes* mosquitoes were concentrated in the city center close to the city hall (Fig. 1b). Temporally, there was a sharp increase in mosquito numbers in the transition from spring to summer and a decrease in the fall (Fig. 1c).

Out of the total, we detected 43 *Aedes* mosquitoes (36 *Ae. aegypti* females*,* two *Ae. aegypti* males and five *Ae. albopictus* females) that were positive for DENV. Infection of male mosquitoes is indicative of DENV circulation between mosquitoes but does not directly contribute to virus transmission to humans. Considering only *Aedes* females, we observed a total DENV prevalence of 5.6%, or 41 individuals out of 728 (Fig. 1d). This number is higher than observed in other studies where DENV prevalence in mosquitoes ranged between 0.58 and 1.8% [38–40]. However, our strategy involved sample preparation and testing of individual mosquitoes, which increases the sensitivity compared to the analysis of mosquito pools in previous studies [41–44].

Regarding the diversity of DENV serotypes, we observed that the majority of wild *Aedes* female mosquitoes carried DENV1 or DENV3 (Fig. 1d). One *A. albopictus* female was positive for DENV4 but was not included in further analysis due to the small numbers. For the subsequent spatio-temporal analyses of DENV transmission, we only considered female *Aedes* mosquitoes infected with DENV1 and DENV3.

### Identification of the parameters of the variograms for the spatial incidence of DENV1 and DENV3 in mosquitoes

**Fig. 2:**
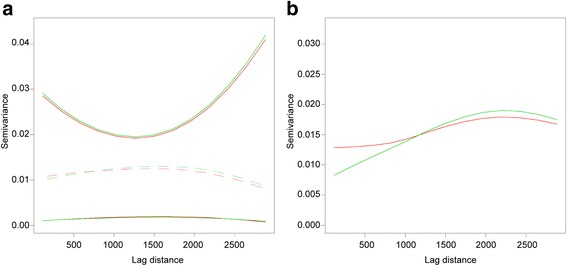
Similarities in the spatial dependence of DENV1 and DENV3 incidence in mosquitoes. **a** Variogram envelopes where dashed lines are the modelled semivariance (variance between points at a varying distance) for DENV1 (*green*) and DENV3 (*red*) incidence in mosquitoes. The continuous lines are the envelopes for DENV1 (*green*) and DENV3 (*red*). If the modelled variogram crosses the simulation envelopes it is considered significantly different which was not the case. **b** Variogram isotropy test where *ΔM,* DENV incidence, variograms at 0 (*green*) and 90 (*red*) degrees. The two curves shows that the variogram changes along distance are similar in the two orthogonal directions in space

We next analyzed the spatial DENV incidence in mosquitoes by fitting a variogram. A variogram is a function of the variance of an attribute between point locations, in this case DENV incidence between trap locations. This variance is usually lower at shorter distances so that closer locations are more likely to have similar values than locations far apart. The variograms of DENV1 and DENV3 incidence in mosquitoes were fitted with a restricted maximum likelihood approach using an exponential function that returned lowest errors in cross validation (via kriging) (mean error 0.0002; mean squared error 0.006). For both DENV1 and DENV3, we also calculated the variogram confidence envelopes, which indicate the amount of variation that would be expected in the case of independence between incidence and location. Figure 2a shows an almost identical autocorrelation pattern and envelopes for the two DENV serotypes (from 1000 permutations).

Since the two variograms are statistically very similar, we merged the data for DENV1 and DENV3 in a single variable, *ΔM.* This new variable contains the values of DENV1 incidence in mosquitoes for humans infected with DENV1, and DENV3 incidence in mosquitoes for humans carrying DENV3; in this manner, we are not averaging the incidence, but unifying them in a single variable. We then tested if this variable is isotropic in space or, in other words, if the variogram changes with direction. Figure 2b shows that the variograms at 0 and 90 degrees are very similar suggesting that an isotropic model (that does not account for direction) can be used for the next analyses.

In summary, our results indicate that the incidence of DENV1 and DENV3 in mosquitoes is characterized by a similar spatial process, which is invariant with direction (North to South and East to West). The common estimated variogram parameters are a spatial range of 1.469 km (1.731 km and 1.261 km at 95% permutation interval), a noise of 0.010 (0.002 and 0.017 at 95% permutation interval) and spatially dependent variance of 0.006 (0.002 and 0.006 at 95% permutation interval). The observed spatial range of 1.469 km is larger than the average dispersal of *Aedes* mosquitoes [25], but lower than the known spatial scales of *Aedes* urban geographical distribution [45].

### Human data collection and analysis

In total, we analyzed 44 human blood samples representing 47% of the dengue patients notified based on symptomatology by the city of Caratinga for the 2010/2011 outbreak. We first analyzed human samples for presence of DENV-specific antibodies (IgM and IgG) and observed that 24 out of the 44 (54.5%) were positive for anti-DENV IgG (Table 1). Detection of DENV specific IgG suggests that the population in Caratinga had previous contact with DENV. Fifteen of these 24 samples were also positive for anti-DENV IgM suggesting a primary response to an ongoing infection. We were able to detect DENV RNA by RT-PCR in 15 out of the 44 blood samples suggesting that the majority of patients did not present detectable viraemia at the time of the sample collection (Fig. 1e). Indeed, viraemia is detectable for only a short period during the early stages of infection, which usually coincides with the appearance of symptoms [46].

**Table 1:** Data summary of human patients

Patient number	Age (years)	Gender	Collection date	Time of collection^b^	DENV type (PCR)	IgM/IgG^c^
1	58	F	02/14/11	13	–	−/−
2^a^	27	M	02/14/11	7	DV1	−/−
3	25	M	02/21/11	11	–	−/−
4	51	F	02/10/11	6	–	−/−
5	32	M	02/14/11	87	–	−/+
6	22	M	02/08/11	3	–	−/+
7	28	M	02/08/11	30	–	−/−
8	32	M	02/02/11	17	–	−/−
9	22	F	02/02/11	24	–	−/+
10^a^	54	M	02/02/11	9	DV1	−/−
11	13	F	02/14/11	7	–	−/−
12^a^	21	F	02/14/11	13	DV1	−/−
13^a^	49	M	03/14/11	25	DV3	−/−
14^a^	39	F	03/14/11	7	DV2	+/+
15^a^	33	F	02/25/11	8	DV1/DV3	+/+
16^a^	35	F	03/07/11	23	DV4	−/+
17^a^	65	M	02/25/11	38	DV1	+/+
18^a^	15	M	02/25/11	7	DV1	+/+
19	15	M	03/07/11	42	–	+/+
20^a^	54	F	03/01/11	44	DV1/DV3	+/+
21^a^	8	F	03/16/11	35	DV1/DV3	+/+
22	32	M	02/09/11	26	–	−/+
23	4	F	02/14/11	7	–	−/−
24	11	F	02/14/11	7	–	−/+
25	0,4	M	02/18/11	8	–	−/−
26^a^	49	F	04/04/11	8	DV1	+/+
27^a^	49	F	03/29/11	30	DV1	+/+
28	20	F	03/24/11	31	–	+/+
29	19	M	04/05/11	13	–	+/+
30	23	F	04/01/11	8	–	+/+
31	62	M	03/24/11	6	–	−/−
32	21	F	03/29/11	8	–	−/−
33	23	F	04/12/11	19	–	−/+
34^a^	28	M	06/13/11	19	DV3	+/+
35	25	F	07/12/11	9	–	−/−
36	10	F	04/20/11	69	–	−/−
37	12	M	04/11/11	–	–	−/−
38	23	M	04/08/11	6	–	−/−
39	56	M	04/04/11	7	–	−/−
40	25	F	04/08/11	11	–	−/−
41	15	M	04/20/11	42	–	−/+
42^a^	46	F	06/13/11	9	DV1	+/+
43	42	F	07/05/11	30	–	+/+
44	19	F	03/24/11	53	–	−/+

^a^Patients used for the present spatio-temporal analyses

^b^Days after the beginning of symptoms

^c^Anti-DENV IgM and IgG

Regarding the diversity of DENV types, DENV1 and DENV3 were detected in the majority of human samples from Caratinga (Fig. 1e). The exception was DENV2 that was detected in one human patient but not in wild mosquitoes. We note that DENV1 and DENV3 were similarly observed in human and mosquito samples (as reported elsewhere [47, 48]). Thus, we then restricted our spatio-temporal analysis of human patient data to the 15 individuals (see the following sections) with confirmed presence of DENV1 and DENV3 by RT-PCR (rows indicated in Table 1).

### Combining human and mosquito data: identification of the spatial and temporal scales of DENV transmission

**Fig. 3:**
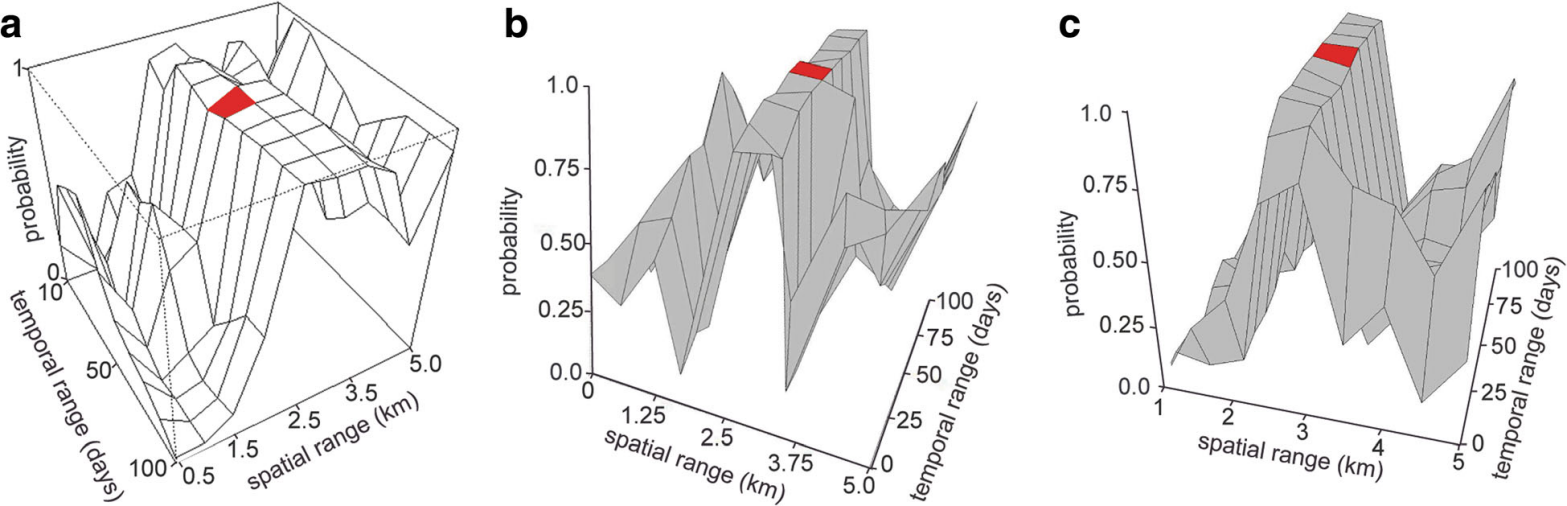
Probability of DENV incidence in mosquitoes around patient locations. We calculated whether the probability that DENV incidence in mosquitoes around patient locations was larger than in other permutation of the locations “before human infection” (**a**), “after human infection” (**b**) or “before and after human infection” (**c**). The highest observed probability (1-*mcp*) was concentrated between 2.5 and 3 km spatial range and 30 to 70 days temporal range, with a maximum at 2.5 km and 50 days (red area) in the model “before human infection”

To identify the spatial and temporal scales of virus transmission we tested if DENV incidence in mosquitoes in the proximity of patient locations was significantly higher at patient locations (as measured by the probability 1 – *mcp*, with *mcp* being the number of permutations with higher dengue incidence in mosquitoes than any other configuration of DENV incidence in mosquitoes). Therefore, we identified the most significant scenario as the one with lowest *mcp* value. This analysis showed that the probability of DENV incidence in mosquitoes was significantly higher within 2.5 km from patient locations and 50 days before the appearance of dengue symptoms in humans (“before human infection”) compared to a random permutation of human cases (*mcp* = 0.2, which means that only 0.2% randomizations returned a higher value of DENV incidence) (Fig. 3a). Notably, other combinations of 2.5 and 3 km with 30 to 70 days “before human infection” returned values of *mcp* lower than 5%, and therefore epidemiologically important. In terms of a possible directionality of transmission, the analysis showed that spatial pattern in human infections was more likely the result of increased incidence of DENV in mosquitoes “before human infection” (which may suggest a flow of DENV from mosquitoes to humans), since a 0.2% *mcp* was lower than 19% *mcp* for “after human infection” (which may represent DENV flow from humans to mosquitoes) and 1.4% “before and after human infection” (representing an increase in DENV incidence in mosquitoes from before to after the human infections) (Fig. 3a-c). In other words, the chance of having higher incidence of DENV in mosquitoes in proximity of patient locations was almost 100 times lower for “after human infection” than “before human infection”. For “after human infection” the optimal spatial and temporal ranges were identical to “before after infection”, but with none of the combinations resulting in an *mcp* < 5% (Fig. 3b). Finally, for “before and after human infection” test, the optimal ranges were 70 days and 2.5 km, with all the combinations between 2.5 km and 50 to 90 days lower than 5% *mcp* (Fig. 3c). In our analysis, we did not differentiate between infected *A. aegypti* and *A. albopictus* females, because the latter represented just a small proportion of the dataset, and the main interest in this work is on DENV transmission independently from the vector species. However, for completeness running the same framework only for *A. aegypti* mosquitoes, we obtained the same optimal spatial and temporal ranges, and spatial credible interval (2.5–3 km), but with larger credible interval for the temporal range (20–70 days) than “before human infection”.

### Modeling the association between environmental variables and DENV incidence in mosquitoes around patient locations: a spatio-temporal additive, geostatistical, linear model

The spatio-temporal modeling of the association between environmental variables and DENV incidence in mosquitoes 50 days before human infections and 2.5 km around patient locations was then analyzed. We note that this analysis is limited by the small sample size since we had to use only data for DENV1 and DENV3 infections in mosquitoes and humans. However, the combinations of mosquito survey sites and human locations at 2.5 km and 50 days, generated a dataset of slightly more than 1000 records.

Our results showed that there were three environmental variables, wind speed (*P*-value of 0.03), wind direction (*P-*value of 10^−7^) and air temperature (*P*-value of 0.002) that significantly correlated with spatiotemporal DENV incidence in mosquitoes in proximity of patient locations (Table 2). The cross validation test for spatio-temporal additive, geostatistical and linear model considering the environmental variables returned a mean error of 0.0008, a mean squared error of 0.003 and a mean squared deviation ratio of 0.999 (ideal value 1). The mean error and mean squared error are 2 and 8% of the incidence mean (0.036), respectively.

**Table 2:** Estimated coefficients for important variables associated with DENV incidence in mosquitoes around patient locations

Model	Coefficients	Estimate	Standard error	*P*-value
DENV incidence in *Aedes* mosquitoes	Intercept	0.210	0.064	0.001
	Wind speed	-0.013	0.006	0.03
	Wind direction	0.003	0.000	10^−7^
	Air Temperature	0.006	0.002	0.002

## Discussion

To our knowledge, this is the first report on the analysis on the spatial and temporal scales of DENV transmission between mosquitoes and humans in a small urban setting. We surveyed individual mosquitoes over a one-year period throughout most of a small urban area and analyzed a significant number of human samples collected during the concurrent dengue outbreak providing important information about the complexity of DENV circulation in mosquitoes and humans. Previous attempts focused on analyzing the clustering pattern of human dengue cases without taking into account vector populations or DENV incidence in mosquitoes (e.g. [49–51]). An exception to this can be found in [52], where the authors investigated the number of human dengue cases per district and the relative mosquito density (but not DENV incidence in mosquitoes), using a generalized linear mixed model. In addition, only a few studies have characterized the distribution of DENV types detected in mosquitoes and humans during the same outbreak [53, 54].

Despite the lack of research in the spatio-temporal scales of DENV transmission, it is well accepted that early warning systems and disease surveillance may benefit from this information [55, 56]. Dengue epidemiological surveillance in Brazil has often taken into account vector density and the number of people infected with DENV [57]. The former is estimated by the Breteau Index (BI), which is the number of positive containers per 100 houses inspected. For the human dengue cases, the WHO suggested the “endemic channel” rule, which identifies an outbreak if the number of cases reported is larger than two standard deviations above the “endemic channel” (weekly or monthly average incidence in the previous 5 to 7 years) in weekly or monthly reports [58]. During an outbreak, a perifocal spraying strategy is often utilized where insecticide is applied around 400 m radius of the residence where a human was detected with dengue. As pointed out by the WHO guidelines, this approach does not account for the time delay between when a person is infective to mosquitoes and the appearance of symptoms. In addition, the large number of asymptomatic cases and the capacity of infected people to transport DENV over longer distances more rapidly than infected mosquitoes pose a serious limitation to the efficacy of perifocal spraying [26]. It is noteworthy that our sampling design and statistical method do not allow us to estimate what proportion of the variance in incidence falls within the 400 m range. However, the spatial range of 1.5 km suggests that transmission occurs more at neighborhood than household level. Therefore, the radius of perifocal spraying should be extended, unless areas at higher risk of infections are known prior to an intervention.

We tested if DENV incidence in mosquitoes around locations where humans were detected with dengue was higher “before human infection”, “after human infection” or “before and after human infection”. We found that the strongest association of an increase in DENV incidence in mosquitoes occurred “before human infection”. The weaker association between DENV incidence in mosquitoes “after human infection” may suggest saturation of the infection in the mosquito population. In addition, vector control interventions after the start of the human outbreak can contribute to suppress DENV incidence in mosquitoes resulting in low values “after human infection”.

Our results indicated a strong spatiotemporal correlation between incidence of DENV in mosquitoes and the appearance of human dengue cases in Caratinga. The approach described in this paper was accurate in identifying the spatial range of transmission but proved less robust in identifying the temporal range. Human infections were strongly associated with higher incidence of DENV in mosquitoes within 2.5–3 km around patient location (optima at 2.5 km) and 30–70 days (optima at 50 days) “before human infection”. Difficulties in sampling infected mosquitoes and detecting infected individuals could have affected our ability to determine a temporal range closer to the extrinsic incubation period, which would be more intuitive. Nevertheless, temporal heterogeneity has been shown in other studies [22]. For example, Pepin et al. [52] found a temporal range of two to 13 weeks between the peak in mosquito density and human infections. Other analyses, focused only on the clusters of human cases, found a temporal lag from 2 to 10 weeks [59, 60]. Certainly, the minimum amount of time necessary from when the a mosquito acquires DENV to the point when it transmits it to a human and the human becoming infectious is generally 2 weeks under favorable climate conditions. However, host-seeking activities and the relative low rate of successful transmission can extend the time lag to well above the two to 3 weeks window [52]. Finally, the temporal accuracy of our results is probably affected by the technique employed for DENV detection in whole mosquitoes, which does not distinguish whether the vector is infectious to humans at the time of collection.

Regarding spatial ranges, we observed 1.5 km for infected mosquitoes and 2.5 km for DENV transmission between mosquitoes and humans. These ranges are larger than some vector distribution and local transmission studies, but are certainly smaller than other medium and large-scale analyses [19, 60]. The distribution of the traps cannot have affected the optimal spatial range since they are homogeneously distributed every ~ 0.2 km^2^ (this accounts for *Aedes* dispersal, estimated to be 800 m every 6 days [61]) and abundant around each patient location (32 to 82 traps) [62]. The size of the urban area, human mobility, asymptomatic cases and vector control interventions (elimination of potential breeding containers, spraying insecticide which tend to disperse the clustered distribution of mosquitoes) could explain why DENV infections in humans are not clustered. The sparsity of human dengue cases is confirmed by the negatively skewed distribution of the spatial ranges towards large values (Fig. 3). All these factors may have contributed to identify a medium-scale of 2.5 km range as most significant [21, 27, 63]. A priori knowledge about household commuting behavior could improve the identification of the spatial range for transmission and help defining zones for targeted control, especially if the scales of human movement exceed that of the vectors.

The ecological analysis (Table 2) confirmed the importance of warm air temperature (in agreement with [64]) and low wind speed in DENV transmission. Indeed, winds are generally an important component in mosquito dispersal and host-seeking strategy [65]. The importance of low wind speed in increasing DENV incidence has been found in Brazil [66, 67] and many other countries including Australia [68], Sri Lanka [69], Barbados [70], Pakistan [71], China [72], Malaysia [73] and Vietnam [74]. In our dataset, wind direction was positively associated to DENV incidence in mosquitoes. This means that winds from the North and Northeast (which are the predominant winds in Caratinga, according to the climatological normal from INMET) were more likely associated with high DENV incidence in mosquitoes. Remarkably, wind direction is the only variable present in all the five best models (in terms of mean error in cross validation), followed by temperature (four out of five best models) and wind speed (two out of five models) (Additional file 1: Table S1). Relative humidity was not selected in the first two models, but has higher frequency (three out of five best models) than wind speed. The total number of mosquitoes caught by the traps was not significant in the best five models.

We also applied a non-spatial model (simple general linear regression) to analyze DENV transmission between mosquitoes and humans. This model showed similar fitting results for wind speed and direction but did not perform better than the spatial model (Additional file 1: Table S1). However, the effect of temperature is inverted (lower temperature associated with larger incidence) due to the fact that the variance is not adjusted for the spatial autocorrelation.

Finally, we found a weak trend (*P* = 0.08) on the question whether infections were simply due to a larger mosquito density around patient locations “before human infection” as suggested by several studies [57, 75]. This result confirms the unclear association between pure vector indices and DENV incidence in humans and reinforces the importance of directly monitoring mosquito infection [76]. However, considering the difficulties in sampling and identifying infected mosquitoes, surveillance based solely on mosquito abundance may still be useful in assessing overall risk, especially when used with other epidemiological parameters.

It is important to point out that our work has some limitations including: (i) small number of samples for human patients and DENV infected mosquitoes; (ii) the fact we did not analyze asymptomatic humans that contribute to DENV circulation and can represent 50–90% of all infections [1]; (iii) the possibility that DENV circulates in other vertebrate hosts [10]; and (iv) the effect of human mobility [27, 63]. Due to the spatial and temporal scale of the DENV epidemic in Caratinga, it is possible that traps with recorded infected mosquitoes, are considered at the same time in different patient locations. Although this is true, we have not found particular traps recurring more often than others. In fact, 60% of the traps are recurring in more than 50% of the patient locations. In addition, the fact that we tested for randomness of spatial and temporal scales within different scenarios (“before human infection”, “after human infection” and “before and after human infection”) has certainly reduced the risk of bias. However, since our analysis only focused on the comparison between scenarios and the accuracy of the obtained parameters, it is possible that additional transmission components, such as human mobility, could significantly change our parameterization.

We observed no evidence that DENV serotypes influence the spatial process (autocorrelation) of infected mosquitoes (Fig. 2a). This result must be confirmed (especially due to the small sample size), since some serotypes may influence mosquito behavior [77]. In contrast, as found elsewhere (e.g. [78]), the distribution of mosquitoes infected with different DENV serotypes is patchy and localized to a small proportion of the territory when the spatial autocorrelation is accounted for.

## Conclusions

Dengue is currently the most prevalent viral disease in the world. Mosquito control strategies can have a significant impact on transmission although vector indices show little correlation with human outbreaks [14]. Thus, better understanding of DENV circulation between mosquitoes and humans in natural settings is required. More accurate estimations of the spatial scales of DENV transmission can help design more efficient surveillance and vector control systems. The method proposed in this work was able to infer parameters of DENV transmission that are coherent with experimental analysis in the laboratory. Our spatio-temporal analysis indicates that local incidence of DENV in mosquitoes is a great indicator of the probability of human dengue cases and should be a major target for surveillance strategies meant to raise preparedness since localized vector control interventions are not likely to work. Altogether, this work contributes to the understanding of the spatial and temporal transmission of DENV in endemic urban areas. The developed framework can be used for any spatiotemporal process with reference points (in statistics, those points containing only the information of presence, like the patient locations) when no initial assumptions about reference (patient) and target (mosquito traps) points can be made.

## Methods

### Study area

The study was conducted in the municipality of Caratinga located in the east of Minas Gerais state, Southeastern Brazil (19°47′24"S, 42°08′20"W). Caratinga has a total territorial extension of 1,258,778 km^2^ with approximately 90,000 inhabitants. Atlantic forest is the biome of the municipality with 21 °C median annual temperature and 80% humidity (data from the Brazilian Ministry of Agriculture, INMET) [79]. Caratinga is a dengue endemic region with 1274, 91, 409 and 51 confirmed human cases each year from 2007 to 2010 prior to this study (data from the Brazilian Ministry of Health, SINAN) [80].

### Mosquito collection

We took advantage of a mosquito monitoring system that has been utilized by Caratinga and other municipalities in the state of Minas Gerais [37]. This system uses mosquito traps known as MosquiTRAP [29, 81] that are placed 300 m apart, following a 300 × 300 m grid design, in a study area. Traps are placed outdoors, usually in the front or backyard of the property where access is restricted. Traps are never placed in open areas accessible to the general public. Whenever the 300 m distance between traps could not be achieved, traps were placed in a distance of less than 300 m, never more than this limit. In Caratinga, 158 MosquiTRAPs were installed at an average density of 5.15 traps/km^2^ (3.2–12.5 traps/km^2^, 95% confidence interval) [29]. These traps covered 14.2 km^2^ of the urban and peri-urban areas of the city of Caratinga (Fig. 1). Between August 2010 and July 2011, traps were inspected by local health authorities once a week and each captured mosquito was collected and visually classified by species and gender [82]. Mosquitoes were individually stored in liquid N_2_ before being processed for RNA extraction using Trizol reagent (Invitrogen, Carlsbad, USA).

### Human blood samples

In order to compare DENV circulation in mosquitoes to human dengue, we obtained blood samples from patients that sought medical attention in the city of Caratinga between February and July of 2011, concomitantly with mosquito captures. Human samples were collected by professional nurses employed by the health system of Caratinga as part of a city surveillance plan. Blood samples were collected at home from patients that had any dengue related symptoms such as fever, anorexia, myalgia, arthralgia and headaches and requested assistance. We obtained 44 samples representing 47% of the 94 patients that sought medical attention by health professionals from the city of Caratinga. Samples were collected at different times after the appearance of symptoms ranging from 3 to 87 days. Blood samples were mixed with EDTA as an anticoagulant and stored at 4 °C. Serum was obtained from blood samples and inactivated at 56 °C for 30 min. Inactivated serum was tested for the presence of DENV specific IgM and IgG utilizing the Panbio Dengue IgM Capture ELISA (Alere, Waltham, USA) and Dengue IgG ELISA Test (HUMAN Diagnostics, Wiesbaden, Germany). For each patient, we utilized information of time and location based the date of onset of symptoms and home address reported to health authorities. Total RNA extraction from human blood samples was performed using Trizol LS Reagent (Invitrogen). Unlinked anonymous testing of human blood samples was approved by the ethics committee in research (COEP) of Universidade Federal de Minas Gerais (number 415/04 to EGK).

### DENV detection by RT-qPCR and RT-PCR

RNA extracted from whole mosquitoes and human blood samples were screened for the presence of DENV RNA by RT-qPCR. For this purpose, we designed specific primers targeting a conserved region in the 5’ UTR of the DENV genome of different virus types (Additional file 2: Table S2). Samples that showed DENV amplification by RT-qPCR were subsequently tested using a previously described nested RT-PCR strategy [83]. This strategy utilizes DENV generic primers on the first PCR followed by a second reaction using primers targeting a region of the NS5 gene that are specific for each virus type (Additional file 2: Table S2). Reverse transcription was performed using total RNA (200 ng to 1 μg) and random primers (300 ng/μl) using MMLV reverse transcriptase. qPCR was performed using Power SYBR Green I (Applied Biosystems, Foster City, USA) and 2 pmol of specific primers. Conventional PCR was performed using 10 pmol of gene specific primers. Importantly, in the case of mosquitoes, our strategy is not able to infer whether individuals are infective at the time of collection.

### Statistical analyses

Statistical analyses comprised: (A) parameterization of the variogram for incidence of each DENV serotype in mosquitoes and analysis of their differences; (B) estimation of the spatial and temporal scales in local DENV transmission and calculation of their significance by 10,000 permutations of patient locations (home addresses of each human case was used as patient location); and (C) spatio-temporal additive, geostatistical, linear model on the incidence of DENV in mosquitoes found around patient locations and using environmental data.

Abundance of adult female mosquitoes is considered the most appropriate measure of entomological risk [26]. Therefore, we did not use two DENV infected and 31 uninfected *Aedes* males in our spatiotemporal analysis. We considered females of *Ae. aegypti* combined with *Ae. albopictus*, since we were more interested in the regional dynamics of DENV transmission and not specific contributions of each vector. However, we recognize that the two vectors have a different ecology and behavior that can affect the spatial and temporal scales of DENV transmission [84, 85]. We compared our results using both mosquito species to *Ae. aegypti* alone and observed no major differences. A separate analysis of *Ae. albopictus* was not possible due to the limited amount of traps with infected (only five found in two traps) and uninfected *Ae. albopictus*, which are predominantly located on the edges of the city.

We used the variogram parameterization from (A) to inform kriging (value of a function at a given point by computing a weighted average of the known values of the function in the neighborhood of the point) in (B). Then we used the spatial and temporal ranges obtained in (ii) to produce the response variable for (C).

#### Parameterization of the variogram

For each mosquito trap we calculated the incidence of DENV in mosquitoes (which is the ratio between number of infected mosquitoes for each DENV serotype and the total number of captured individuals in a trap in a week). DENV incidence in mosquito variogram parameters (temporal range, *t*, and spatial range *s*) are fitted with a restricted maximum likelihood approach [86] using a spatio-temporal exponential function, where *d* and *h* are the spatial and temporal Euclidian distances, respectively, between trap locations.

$$ \widehat{\gamma}\left(s,t\right)=\kern0.5em \exp \left(-\frac{d}{s}\kern0.5em +\frac{d}{s}\kern0.5em \frac{h}{t}-\frac{h}{t}\kern0.5em \right) $$

The exponential function form was chosen since it returned the lowest errors in cross validation (via kriging) compared to spherical and Gneiting functions [87]. We tested if there were any differences in the spatio-temporal process (in terms of variogram parameterization) of the incidence for each DENV serotype in mosquitoes. To evaluate this, we permuted data values of DENV incidence at the spatial locations to produce upper and lower envelopes of the variogram for each virus type [88, 89]. We then assumed that the variograms of DENV serotypes are identical if the area within the lower and upper envelopes overlap by 95%. Finally we explored the presence of anisotropy (i.e. when the spatial variation around each point have a component of direction) by plotting the variograms at different directions [90].

#### Spatial and temporal scales in local DENV transmission and their statistical significance

The spatial and temporal ranges of DENV transmission were estimated by finding those scales that maximize the predicted mean incidence of DENV in mosquitoes at the patient location, i.e. the geographical coordinates at the exact home address, compared to the predicted mean from a set of permutations of patient locations. In practice:

1. Define a grid of spatial and temporal ranges.
2. For each spatial and temporal range combination:
  - For each patient location, select those mosquito traps (and relative DENV incidence) within the selected spatial and temporal ranges around the patient, and considering only the mosquitoes infected with the same DENV serotype detected in the patient.
  - For each patient location, *v*, apply local ordinary kriging [using the parameterization obtained from section (A)] to the DENV incidences selected in the previous step to estimate the value of incidence, ${\widehat{I}}_v$ (kriging applied individually). The mean of the estimated values, *F1*, is:

$$ F1=\frac{1}{N}{\sum}_{v=1}^N{\widehat{I}}_v $$

where N is the total number of patients.

c. Create a variable “count” equal to 0.
d. Repeat for 9999 times:

i. Permute the location of human patients in time (e.g. swapping the time of human infection between locations)
ii. Recalculate (a) and (b)
iii. If the new mean of the kriging fitted values is larger than F1, then add 1 to count.

e. Calculate *mcp*, the probability that DENV incidence in mosquitoes in permuted locations is higher than the one at the original patient locations:

$$ mcp=\mathrm{count}/10,000 $$

f. Go to (a) with a new spatial and temporal range combination. If all the spatial and temporal range combinations are explored, then stop.

A lower *mcp* indicates lower probability that there is an alternative spatio-temporal configuration of the human patient locations with larger DENV incidence in mosquitoes. This algorithm was repeated for temporal ranges before the human infection (thereafter “before human infection”), after human infection (thereafter “after human infection”) or both (thereafter “before and after human infection”).

Preliminary analyses on mosquito spatial and temporal variogram restricted the exploration of spatial ranges between 0.5 and 5 km and temporal ranges between 0 and 100 days [30]. These spatial and temporal ranges are at the low and high limits expected for mosquito-human interactions in DENV transmission [52]. Finally mosquito DENV incidence within the optimized spatial and temporal range from human patient location, *ΔM*, were selected as response variable for the next step of the analysis.

#### Spatio-temporal additive, geostatistical, linear model on the incidence of DENV incidence in mosquitoes around human patient locations

An additive, geostatistical, linear model was applied to the incidence of DENV in mosquitoes at survey locations in proximity (in space and time) of infected patient locations, *ΔM*, as described in [30] but without autoregressive term and random effects. The additive, geostatistical, linear model contains a matrix of covariates **X** (fixed effects); a spatiotemporal correlation effect, **Z**; and an error component *ε*:

$$ \Delta {M}_{q,u}=\beta X+Z+\varepsilon $$

$$ Z\sim N\left(0,{\sigma}_z^2\widehat{\gamma}\left(s,t\right)\right) $$

$$ \varepsilon \sim N\left(0,{\sigma}_{\varepsilon}^2I\right) $$

therefore:

$$ \Delta {M}_{q,u}\sim N\left(\beta X,{\sigma}_z^2\widehat{\gamma}\left(s,t\right)+{\sigma}_{\varepsilon}^2I\right) $$

where the subscripts *q* and *u* indicate the location and the time of the patient event, respectively; *β* is a vector of coefficients for **X**; **Z** is a one column vector with spatio-temporal normally distributed random effects with mean zero and a covariance matrix given by the product of the spatial variance, ${\sigma}_z^2$, and the correlation matrix, $\widehat{\gamma}$. As shown above, $\widehat{\gamma}$ is expressed as a function of the spatial correlation parameter, *s*, defining the spatial range of *ΔM*; and the temporal correlation, *t*, defining the temporal range of *ΔM*. Finally, *ε* is the independent and identically normally distributed error, with error variance ${\sigma}_{\varepsilon}^2$**I**. The candidate covariates in **X** are total number of mosquitoes, temperature, atmospheric pressure, wind speed, wind direction, and relative humidity. Important variables were selected based on the improvement of the mean standard error in cross-validation from the total 63 possible combinations between variables.

Model validation was performed by leave-one-out cross validation, for which we produced three statistics: the mean error, the mean squared error and the mean squared deviation ratio (full description in [30]).

## Additional files

Additional file 1: Table S1. Selection results for linear and non-linear models. (PDF 76 kb)

Additional file 2: Table S2. Oligonucleotides primers utilized for PCR amplification of specific targets. (PDF 91 kb)

## Abbreviations

DENV: Dengue virus
EDTA: Ethylenediaminetetraacetic acid
ELISA: Enzyme-linked immunosorbent assay
IgG: Immunoglobulin G
IgM: Immunoglobulin M
RNA: Ribonucleic acid
RT-PCR: Reverse transcription polymerase chain reaction
