# Supplementary material for: The spatial and temporal scales of local dengue virus transmission in natural settings: a retrospective analysis
Source: Parasit Vectors. 2018 Feb 2;11:79. doi: 10.1186/s13071-018-2662-6 (PMC5797342; doi:10.1186/s13071-018-2662-6)
Supplement: Supplementary file 1 — Selection results for linear and non-linear models. (PDF 76 kb) [file 13071_2018_2662_MOESM1_ESM.pdf]

Additional File 1

The spatial and temporal scales of local dengue virus transmission in natural settings: a retrospective analysis

Luigi Sedda, Ana Paula Pessoa Vilela, Eric Roberto Guimarães Rocha Aguiar, Caio Henrique Pessoa Gaspar, André Nicolau Aquime Gonçalves, Roenick Proveti Olmo, Ana Teresa Saraiva Silva, Lízia de Cássia da Silveira, Álvaro Eduardo Eiras, Betânia Paiva Drumond, Erna Geessien Kroon and João Trindade Marques*

*Correspondence: jtm@ufmg.br

Table S1. Selection results for linear and non-linear models[a]

Rank	Total number of mosquitoes	Wind direction	Wind speed	Temperature	Relative humidity	Atmospheric pressure	Mean squared error[b]
1		0.0031 (1.0e-7)	-0.0131 (0.03)	0.006 (0.002)			0.00423
2		0.0021 (1.1e-04)		0.0004 (0.81)		-0.0001 (6.2e-06)	0.00509
3		0.0039 (2.1e-11)	-0.0434 (2.1e-11)	0.0047 (0.01)	-0.0038 (1.1e-09)		0.00519
4		0.0025 (1.4e-06)			-0.0015 (0.003)		0.00577
5		0.0024 (1.2e-05)		0.0026 (0.14)	-0.0017 (1.2e-03)		0.00647
Non spatial		0.0011 (1.0e-6)	-0.019 (1.0e-4)	-0.009 (1.0e-5)			0.00561

[a] Models are ordered from rank 1 to 63 based on their mean squared error obtained in cross validation. Only the top 5 are shown here. The table reports the model rank, the estimated regression coefficients and p-values (in brackets) for mosquito count (in none of the 5 best models mosquito count was selected), wind direction, wind speed, temperature and atmospheric pressure. The last column shows mean squared errors for each model. The last row reports the result for the best non-spatial model.

[b] Mean error is calculated from cross validation.
