# Supplementary material for: The spatial and temporal scales of local dengue virus transmission in natural settings: a retrospective analysis
Source: Parasit Vectors. 2018 Feb 2;11:79. doi: 10.1186/s13071-018-2662-6 (PMC5797342; doi:10.1186/s13071-018-2662-6)
Supplement: Supplementary file 2 — Oligonucleotides primers utilized for PCR amplification of specific targets. (PDF 91 kb) [file 13071_2018_2662_MOESM2_ESM.pdf]

Additional File 2

The spatial and temporal scales of local dengue virus transmission in natural settings: a retrospective analysis

Luigi Sedda, Ana Paula Pessoa Vilela, Eric Roberto Guimarães Rocha Aguiar, Caio Henrique Pessoa Gaspar, André Nicolau Aquime Gonçalves, Roenick Proveti Olmo, Ana Teresa Saraiva Silva, Lízia de Cássia da Silveira, Álvaro Eduardo Eiras, Betânia Paiva Drumond, Erna Geessien Kroon and João Trindade Marques*

*Correspondence: jtm@ufmg.br

Table S2. Oligonucleotide primers utilized for PCR amplification of specific targets.

Target	Organism	Name	Sequence
**DENV - 5' UTR region – qPCR**	DENV4	DV-4UL	TCGGAAGCTTGCTTAACACA
		DV-4UR	TTCGTTGGTTCATTTTTCCAG
	DENV2	DV-2UL	AAGCTCAACGTAGTTCTAACAGTTTTT
		DV-2UR	GCCTTTTTCCGTTGGTTATTC
	DENV1/DENV3	DV-1/3UL	TCGGAAGCTTGCTTAACGTAG
		DV-1/3UR	TCCGTTGGTTGTTCATCAGA
**DENV - NS5 region – conventional PCR**	Generic DENV	FG1 - Left	TCAAGGAACTCCACACATGAGATGTACT
	Generic DENV	FG2 - Right	GTGTCCCATCCTGCTGTGTCATCAGCATACA
	DENV1	ND1 - Right	CGTTTTGCTCTTGTGTGCGC
	DENV2	ND2 - Right	GAACCAGTTTGTTTDRTTTCATAGCTGCC
	DENV3	ND3 - Right	TTCCTCGTCCTCAACAGCAGCTCTCGCACT
	DENV4	ND4 - Right	GCAATCGCTGAAGCCTTCTCCC
***Aedes* ribossomal protein L32 (RpL32) - qPCR**	*A. aegypti* and *A. albopictus*	A.aegL32*-43-Left	AGCCGCGTGTTGTACTCTG
		A.aegL32*-112-Right	ACTTCTTCGTCCGCTTCTTG
